# Reddening of the Unicellular Green Alga *Euglena gracilis* by Dried Bonito Stock and Intense Red Light Irradiation

**DOI:** 10.3390/plants13040510

**Published:** 2024-02-12

**Authors:** Kyohei Yamashita, Ryusei Hanaki, Ayaka Mori, Kengo Suzuki, Tatsuya Tomo, Eiji Tokunaga

**Affiliations:** 1 Department of Physics, Faculty of Science Division I, Tokyo University of Science, 1-3 Kagurazaka, Shinjuku-ku, Tokyo 162-8601, Japan; 1222710@ed.tus.ac.jp (A.M.); eiji@rs.tus.ac.jp (E.T.); 2 Graduate School of Science, Tokyo University of Science, 1-3 Kagurazaka, Shinjuku-ku, Tokyo 162-8601, Japan; 1721521@alumni.tus.ac.jp (R.H.); tomo@rs.tus.ac.jp (T.T.); 3 Euglena Co., Ltd., 1-6, Suehiro-cho, Tsurumi-ku, Yokohama 230-0045, Kanagawa, Japan; suzuki@euglena.jp

**Keywords:** *Euglena gracilis*, carotenoid, bonito, red light, photosynthesis, photoprotection, HPLC, microspectroscopy, integrating sphere, *Euglena sanguinea*

## Abstract

This study confirms for the first time that the significant red coloration of *Euglena gracilis* is induced by bonito stock (BS), a traditional Japanese food, and intense red light exposure (605~660 nm, 1000~1300 µmol photons/m^2^/s). Under the condition, excessive photosynthetic activity destroyed many chloroplasts, while carotenoids were maintained, resulting in the formation of reddened cells. The HPLC analysis revealed that diadinoxanthin was the primary carotenoid present in reddened cells. Additionally, an undefined xanthophyll, not produced under normal culture conditions, was synthesized and suggested to contain a C=O bond. While it has been reported that strong light stress can increase the total carotenoid content of cells, this study did not verify this claim, and it should be investigated further in future research. Under white light irradiation conditions (90 μmol photons/m^2^/s) in BS medium, no reddening of cells was observed, and good growth was achieved (over four times the cell density in CM medium on the seventh day). This cell suspension is considered to have a high nutritional value because it is composed of functional food, BS and *E. gracilis*. The fact that this method does not involve genetic modification suggests the possibility of industrial applications, including food use, even in reddened cells.

## 1. Introduction

Carotenoids have been long utilized as food colorants and antioxidants, as exemplified by *β*-carotene [1,2]. Certain carotenoids might also diminish the risk of cancer and eye diseases [3]. They are employed as additives in animal feed [4], and as active ingredients in cosmetics [5]. In 2022, the worldwide market size for carotenoids reached USD 1.6 billion. The market is anticipated to expand at a compound annual growth rate (CAGR) of 3.6% from 2023 to 2028, culminating in a value of USD 2 billion by 2028 [6].

Carotenoids are biosynthesized in photosynthetic organisms like plants and algae and possess diverse functions related to the utilization of light, such as photosynthesis and photoprotection [7]. The production of carotenoids in microalgae is diverse across different species. Promising microalgae for carotenoid production are those with high production efficiency and the ability to produce healthy carotenoids. Several microalgae species are commonly used, including *Haematococcus pluvialis* [8], *Chlorella* [9], *Spirulina* [10], and *Dunaliella salina* [11]. To achieve highly efficient carotenoid production, genetic engineering techniques are used to select and develop high-carotenoid-producing strains. Strategies to improve production efficiency also include optimizing growing conditions, such as optimizing the medium, adjusting light irradiation, and optimizing nutrient supply [12].

We are focusing on the carotenoid-producing ability of *Euglena gracilis*, an edible unicellular green alga. *E. gracilis* is known to store paramylon, in addition to nutrients suitable for human intake like amino acids, vitamins, and lipids [13,14,15,16]. Paramylon is suggested to have immunomodulatory effects [17], potential preventive effects against atopic dermatitis [18], and antimicrobial effects [19]. Moreover, *E. gracilis* is among the few beneficial organisms capable of producing carotenoids simultaneously with these nutrients [20].

The primary carotenoids in *E. gracilis* are diadinoxanthin, diatoxanthin, neoxanthin, and *β*-carotene [21]. In *E. gracilis*, carotenoids are crucial for photosynthesis (as components of light-harvesting pigment-protein complexes) and photoprotection (encompassing non-photochemical quenching [22], the xanthophyll cycle [21], etc.) [23,24,25]. Also, zeaxanthin, a type of carotenoid, is essential for the stable formation of the eyespot and accurate phototactic responses in *E. gracilis* [26]. Many aspects of the carotenoid-mediated light environment adaptation mechanisms remain unknown [7], and the biosynthetic pathways of carotenoids are also under investigation [27,28].

Aiming to expand the industrial use of *E. gracilis* for food purposes, we have established a spectroscopic method for identifying carotenoids and hematochrome-like granules (structures resembling the eyespot but suggested not to contain carotenoids [29]) within single cells [30]. Additionally, we have developed an efficient cultivation method by growing it in foods, such as tomato juice beverages [31]. Building on these studies, we found that the carotenoid content in *E. gracilis* significantly increased when provided with nutrients from food (such as Katsuo dashi, a traditional Japanese fish stock) and an appropriate light environment (strong red light irradiation). This led to a remarkable reddening at both the culture medium and cellular levels.

*E. gracilis* can grow on heterotrophic culture mediums like Koren–Hutner (KH) medium [32]. Additionally, as the cells contain about ten chloroplasts [33], they can also grow photosynthetically on autotrophic mediums like Cramer–Myers (CM) medium [34]. Beyond these typical synthetic growth mediums, studies have been conducted using food and its by-products as substrates for the growth and upcycling of *E. gracilis*. These studies employed plant-based foods (tomato juice [31], spent tomato byproduct (STB) [35], potato liquor [36], corn steep solid [37], corn steep liquor and brewer’s spent grain [38], kinnow peel extract [39], tofu wastewater [40], and a mixture of sewage and organic wastes (molasses, corn steep liquor, and waste wine) [41]). Our research is the first to use an animal-based food (Katsuo dashi; Bonito stock) in this context.

Bonito stock (Katsuo dashi) is a soup stock extracted from katsuobushi (a traditional Japanese food made from smoked bonito). Dashi, in general, refers to a broth extracted from natural ingredients using water. Its components include amino acids, peptides, inosinic acid, alkaline substances, and trace vitamins and minerals, with sodium inosinate contributing to its renowned umami taste [42,43]. Bonito stock, known as Katsuo dashi, is a functional food with antioxidant [44] and anti-anxiety effects [43].

Previous studies that confirmed an increase in carotenoid synthesis in *E. gracilis* under intense light irradiation and low-temperature stress yielded a yellowish-green culture liquid [21,45]. In contrast, the culture liquid and cells reddened by our method were a reddish-orange color (hereinafter, these altered cells will be referred to as “reddened cells”). To measure the absorbance of this reddened culture liquid and individual cells, we employed a uniquely developed non-scanning absorption spectroscopy imaging method [46]. Furthermore, to mitigate the effects of cellular scattering, we measured the absorption spectrum, including the ultraviolet range, using a spectrometry method that places samples inside an integrating sphere [47]. We also performed high-performance liquid chromatography (HPLC) analysis for the compositional analysis of photosynthetic pigments containing red substances.

A notable species of *Euglena* that exhibits conspicuous cell reddening is *Euglena sanguinea*. *E*. *sanguinea*, in response to light stress, changes from green to red in less than 10 min, not through a time-consuming carotenoid synthesis, but rather through a dynamic photoprotective mechanism based on the movement of cellular organelles containing astaxanthin [48]. This photoprotective mechanism is absent in *E. gracilis*, and there have been no reports of significant reddening in it. Additionally, *E*. *sanguinea* produces toxic alkaloids [49], making it unsuitable for food use.

Hence, our method forms the basis for preferentially producing edible carotenoids in *E. gracilis* by adjusting components derived from food and the light environment, without resorting to genetic manipulation. Moreover, elucidating the reddening phenomenon through this technique contributes to the understanding of the carotenoid biosynthesis pathway, thereby aiding fundamental research as well.

## 2. Results

### 2.1. Exploration of Conditions for Cell Reddening

**Figure 1:**
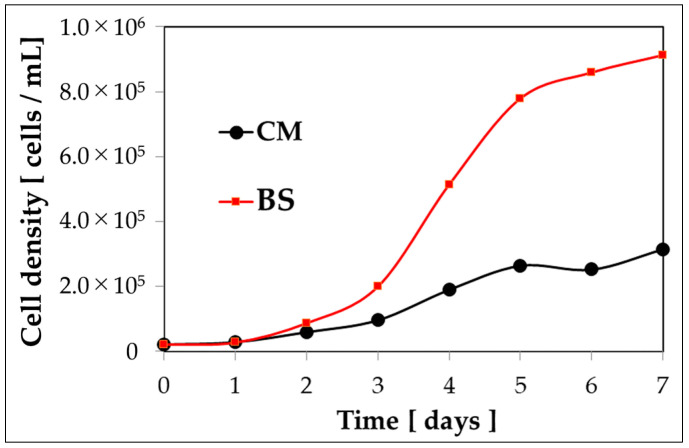
Growth curve in bonito stock culture medium (pre-cultivation for Figure 2). CM: CM medium; BS: Bonito stock medium.

**Figure 2:**
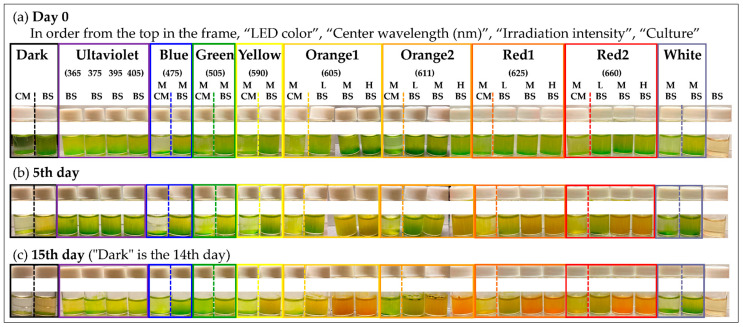
Effect of wavelength and light intensity on cell reddening in CM and BM media. Each symbol corresponds to Table 2. The number of days elapsed excludes the pre-cultivation period (6 days, Figure 1).

For *E. gracilis* cells to undergo reddening, it is necessary to have appropriate conditions in three aspects: composition of the culture medium, wavelength of the irradiation light, and amount of light. Firstly, *E. gracilis* was cultivated under normal light intensity using fluorescent lamps in both BS and CM culture media for 7 days (Figure 1). Separate experiments have been conducted regarding the growth curve up to the stationary phase, and details can be found in the Supplementary Information (Figure S1). The samples grown up to this stationary phase are the control samples, and their cultivation conditions are described in the section “Cultivation of Control Sample”. Subsequently, the wavelength and amount of the irradiation light source were altered for observation over time (Figure 2, Table 1).

During the 7-day cultivation, a four-fold higher cell proliferation rate and cell density were observed in BS medium compared to CM medium (Figure 1). Subsequently, the progress of cultivation over 15 days was observed under the conditions specified in Table 2 (Figure 2). On the 15th day, reddening of the culture medium was observed in samples exposed to warm-wavelength lights “Orange1 (605 nm) to Red2 (660 nm)” with a high amount of irradiation light (“M”, “H”). Additionally, while the BS medium exhibited an orange hue, the CM medium showed green to yellow colors.

### 2.2. Confirmation of Reddening through Absorption Spectra

#### 2.2.1. Absorption Spectrum of Cell Suspensions

**Figure 3:**
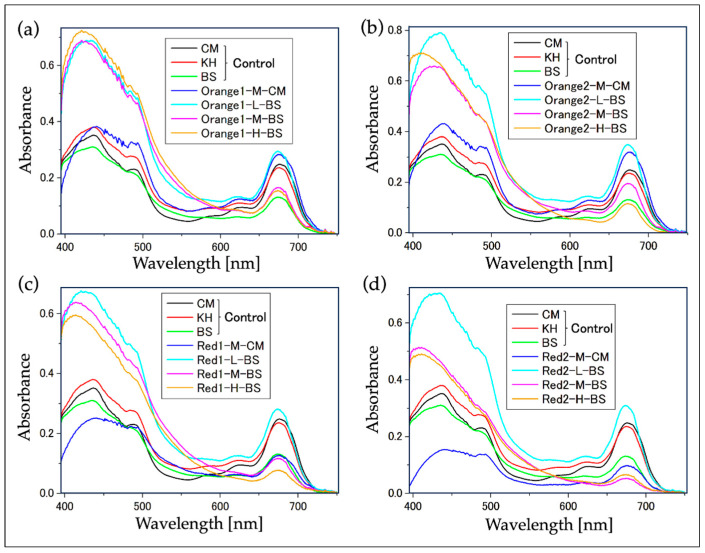
Absorption spectra of cell suspensions at wavelengths where reddening was observed (Figure 2c, 16th day). (**a**) Sample irradiated with “Orange1” LED; (**b**) sample irradiated with “Orange2” LED; (**c**) Sample irradiated with “Red1” LED; (**d**) sample irradiated with “Red2” LED. The control sample (CM, KH, BS) was from a separate set of experiments (see the section titled “Cultivation of Control Sample”) and not part of the series of experiments (Figure 2, Table 2). Symbols for other samples correspond to Figure 2, Table 2. The dilution ratio of the sample solutions varies for each.

**Figure 4:**
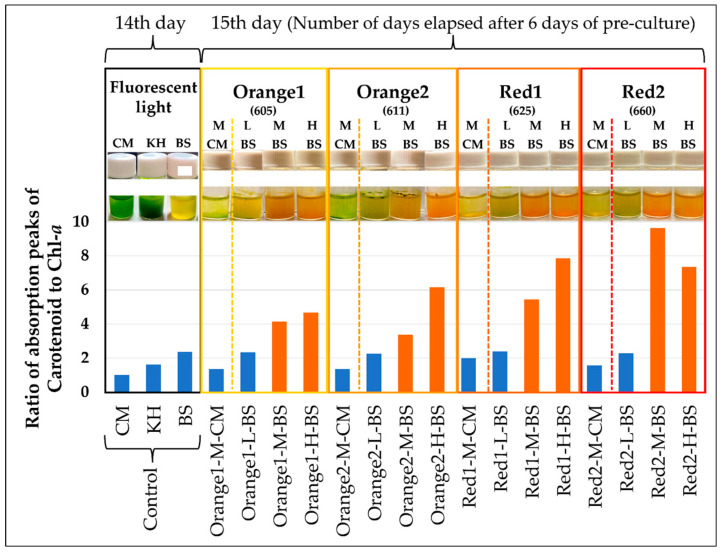
Ratio of absorption peak intensities in culture media (“Carotenoid/Chl-*a*” in Figure 3). Red bars mean samples that were reddened, and blue bars mean samples that were not The Japanese notation in the figure has been removed. The Japanese notation in the figure has been removed. Each symbol corresponds to Figure 2 and Table 2. The control sample (CM, KH, BS) was from a separate set of experiments (see the section titled “Cultivation of Control Sample”) and not part of the series of experiments (Figure 2, Table 2). Symbols for other samples correspond to Figure 2, Table 2.

**Figure 5:**
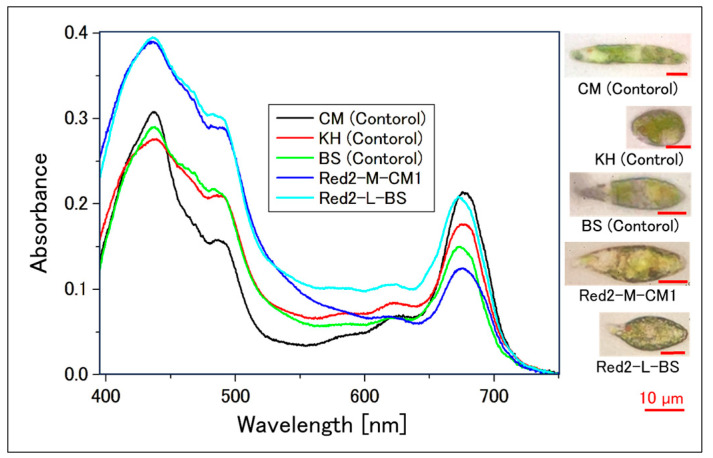
Absorption spectra of single cells (Figure 1). The control sample (CM, KH, BS) was from a separate set of experiments (see the section titled “Cultivation of Control Sample”) and not part of the series of experiments (Figure 2, Table 2). Symbols for other samples correspond to Figure 2, Table 2. Because the photos were taken in dark conditions, the images (CM, KH, BS) have been corrected using the “auto-correction” function of the “Microsoft Office Picture Manager (included in Microsoft SharePoint Designer 2010)”.

**Figure 6:**
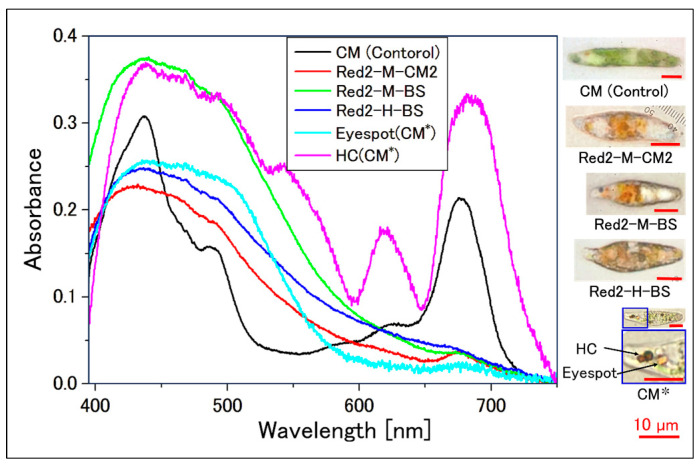
Absorption spectra of single cells (Figure 1). HC: Haematochrome-like granules. The control sample (CM) was from a separate set of experiments (see the section “Cultivation of Control Sample”) and not part of the series of experiments (Figure 2, Table 2). Symbols for other samples correspond to Figure 2, Table 2. CM * (HC, Eyespot) data has been re-edited from our previously published article [23] (photograph at the bottom). Because the photos were taken in dark conditions, the images (other than CM*) have been corrected using the “auto-correction” function of the “Microsoft Office Picture Manager”.

**Figure 7:**
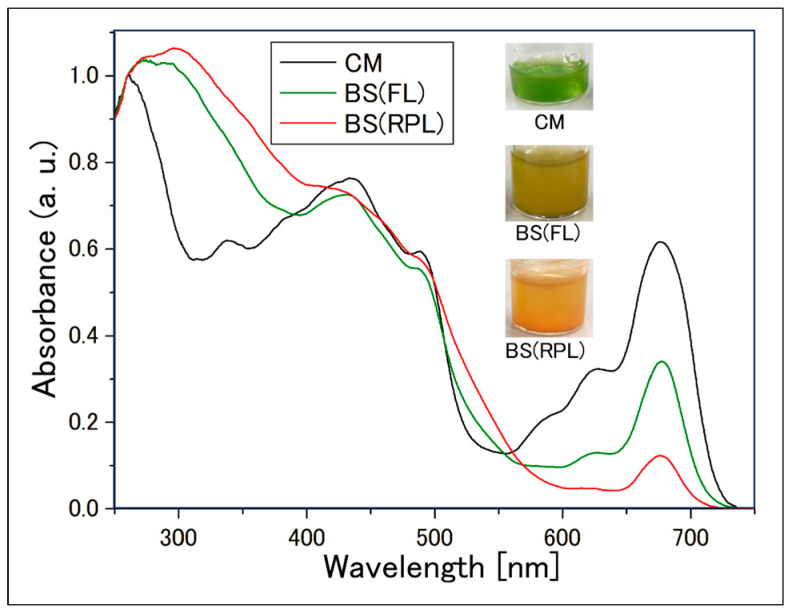
Absorption spectrum of culture media placed inside an integrating sphere. CM: CM medium and fluorescent light (90 µmol photons/m^2^ s). BS(FL): BS* and fluorescent light (90 µmol photons/m^2^ s). BS(RPLE): BS* and Red Power LED (625 nm, 1300 µmol photons/m^2^ s). Vertical axis: Normalized such that the absorbance at 260 nm is 1. For details on the BS*, see Table 3.

In the samples where the culture medium reddened at the wavelengths indicated in Figure 2, the absorption spectrum of the cell suspension was measured (Figure 3), and a comparison of the absorption peaks of carotenoids against Chl-*a* was conducted (Figure 4). The absorption spectrum of individual cells from the aforementioned samples was also measured (Figure 5 and Figure 6). Additionally, absorption measurements extending to ultraviolet wavelengths were carried out using an integrating sphere for both the cell suspensions grown under red light from the outset in bonito stock medium and those grown under white fluorescent light (Figure 7).

From Figure 3, it was observed that the spectrum of the reddened cell suspensions (culture medium “BS”, light amount “M” or “H”) exhibited a markedly smaller absorption peak of Chl-*a* (at 680 nm) relative to the carotenoid peak (around 420 nm).

Regarding the above, from Figure 4, the ratio of absorption peak intensities in the cell suspension (“Carotenoid/Chl-*a*” in Figure 3) in the reddened cell suspensions was around 4~10, and there was a tendency for this value to increase with the irradiation of longer wavelength LED light. On the other hand, in other samples that did not show significant reddening, this ratio was around 2.

#### 2.2.2. Absorption Spectrum of a Single Cell

Figure 5 and Figure 6 show the absorption spectra of single cells frequently found in the cell suspensions in Figure 3d. For microscopic observations of each sample, please refer to the video in the Supplementary Information (Video S1).

Figure 5 shows the absorption spectrum of cells that did not undergo reddening. This spectrum has a shape similar to that of the control samples (CM, KH, and BS).

Figure 6 presents the absorption spectrum of reddened cells. Regardless of the type of culture medium and light amount, the absorption peak of Chl-*a* (680 nm) is significantly smaller in height compared to the control, with a broad absorption peak (420~500 nm) presumably originating from carotenoids. Furthermore, when compared with the absorption spectra of the “Eyespot” and “Haematochrome-like granules (HCs)” within *E. gracilis* cells [30], the spectrum of the reddened cells showed a similarity to that of the “Eyespot”, which predominantly contains carotenoids.

#### 2.2.3. Measurement of Absorption Spectrum of Cell Suspensions Using an Integrating Sphere

Figure 7 shows that in the absorption spectrum in the UV wavelength region, the cell suspensions cultured in BS (“Fluorescent” and “Red PLED”), regardless of the irradiation light wavelength, showed a higher absorption at 280~370 nm compared to the cell suspension “CM” in CM medium. The size of the Chl-*b* absorption peak (620 nm) showed a significant decrease in the reddened cell suspension (Red PLED). The shape of the UV wavelength range absorption spectrum for “CM” was similar to the results of previous studies [50]. In this study, by placing the sample inside an integrating sphere, it was possible to measure the absorption with reduced effects of scattering by the sample [47].

### 2.3. HPLC Analysis of Reddened Cells

**Figure 8:**
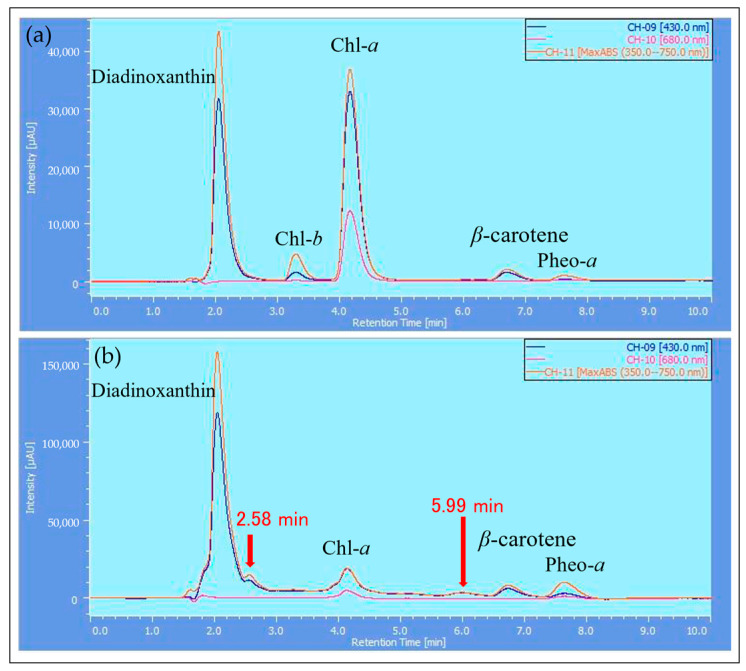
Two-dimensional chromatograms from HPLC (Condition1). (**a**) Cells cultivated under typical growth irradiation light intensity in CM medium. (**b**) Cells reddened by strong red light irradiation in BS medium. The vertical axis in the upper diagram corresponds to absorbance, and the observation wavelength for the upper diagram is shown in the inset at the top right. X-axis: elution time [min]. HPLC (Condition1): MeOH extraction.

**Figure 9:**
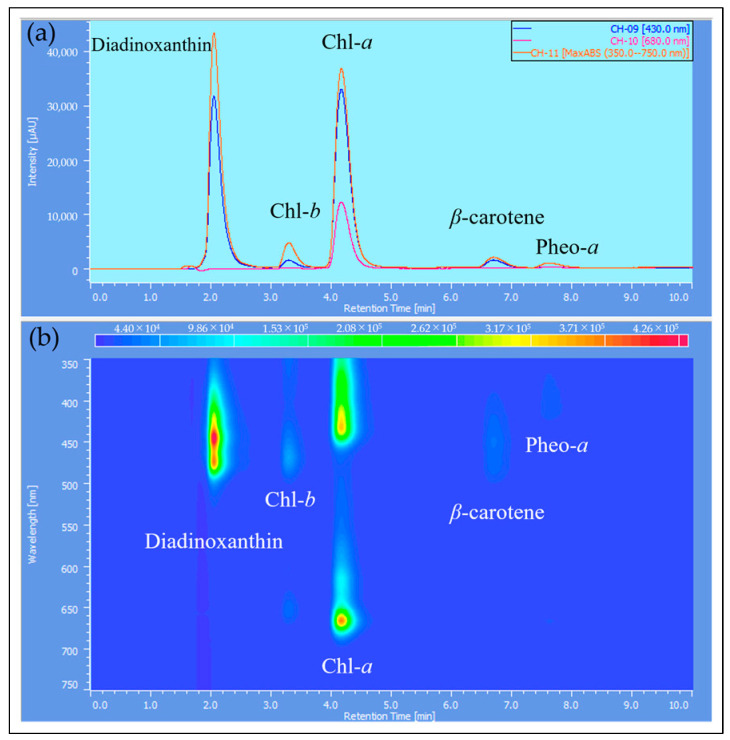
Detailed elution profile from HPLC of cells grown in CM medium (Figure 8a): (**a**) 2D Chromatogram: the Y-axis corresponds to absorbance, and the observation wavelength for the upper diagram is shown in the inset at the top right; (**b**) 3D Chromatogram: the Y-axis represents wavelength, and the Z-axis direction indicates absorbance changes through color variation. X-axis: elution time [min].

**Figure 10:**
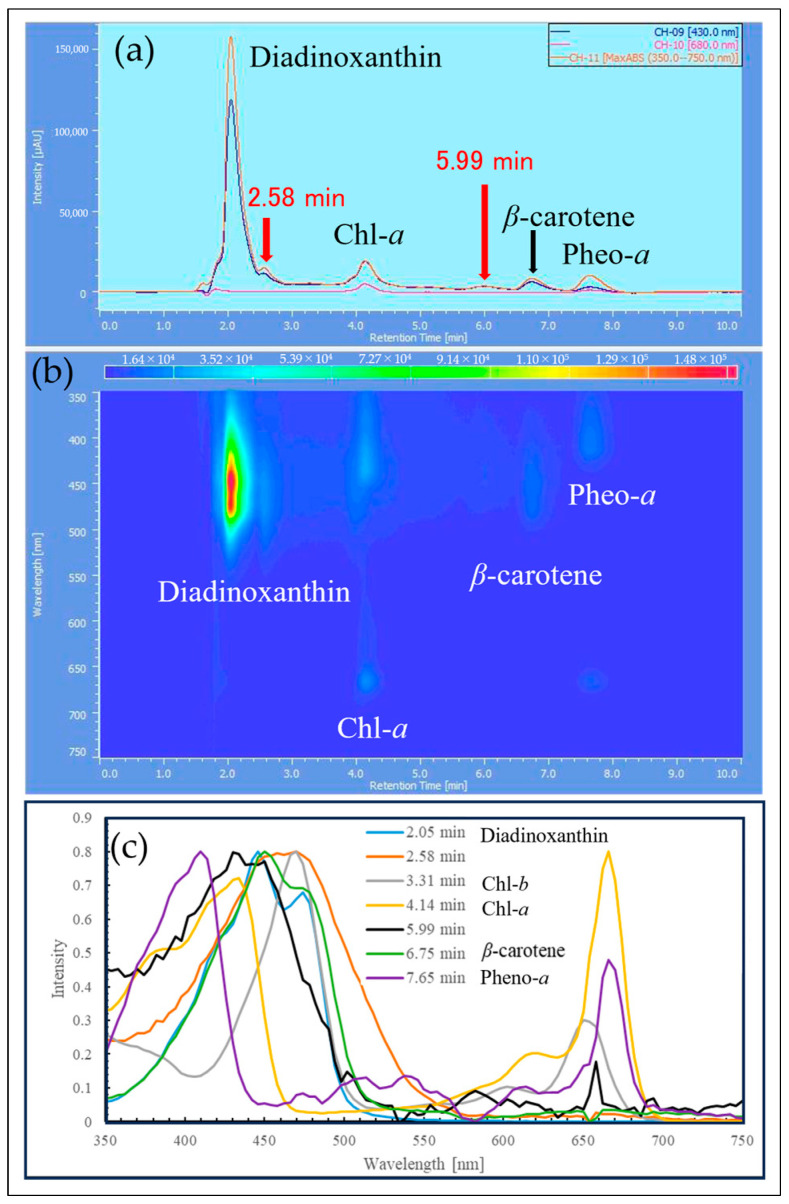
Detailed elution profile from HPLC of reddened cells (Figure 8b): (**a**) 2D chromatogram: the vertical axis in the upper diagram corresponds to absorbance, and the observation wavelength for the upper diagram is shown in the inset at the top right; (**b**) 3D chromatogram: the vertical axis represents wavelength, and the Z-axis direction indicates absorbance changes through color variation; (**c**) absorption spectra and assignments for peaks corresponding to elution times. X-axis: elution time [min].

**Figure 11:**
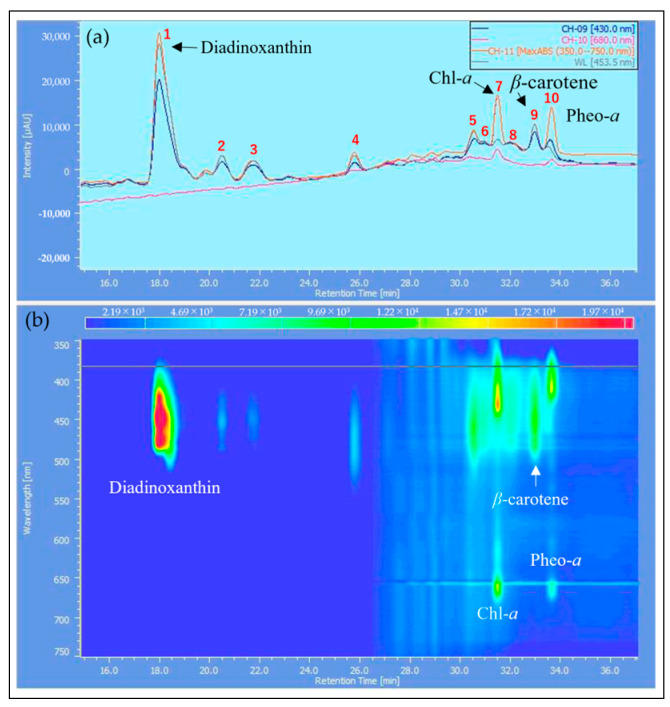
Elution profile from HPLC of reddened cells (Condition2), MeOH extraction: (**a**) 2D chromatogram: the vertical axis in the upper diagram corresponds to absorbance, and the observation wavelength for the upper diagram is shown in the inset at the top right; (**b**) 3D chromatogram: the vertical axis represents wavelength, and the Z-axis direction indicates absorbance changes through color variation. X-axis: elution time [min]. HPLC (Condition2): Zapata method.

**Figure 12:**
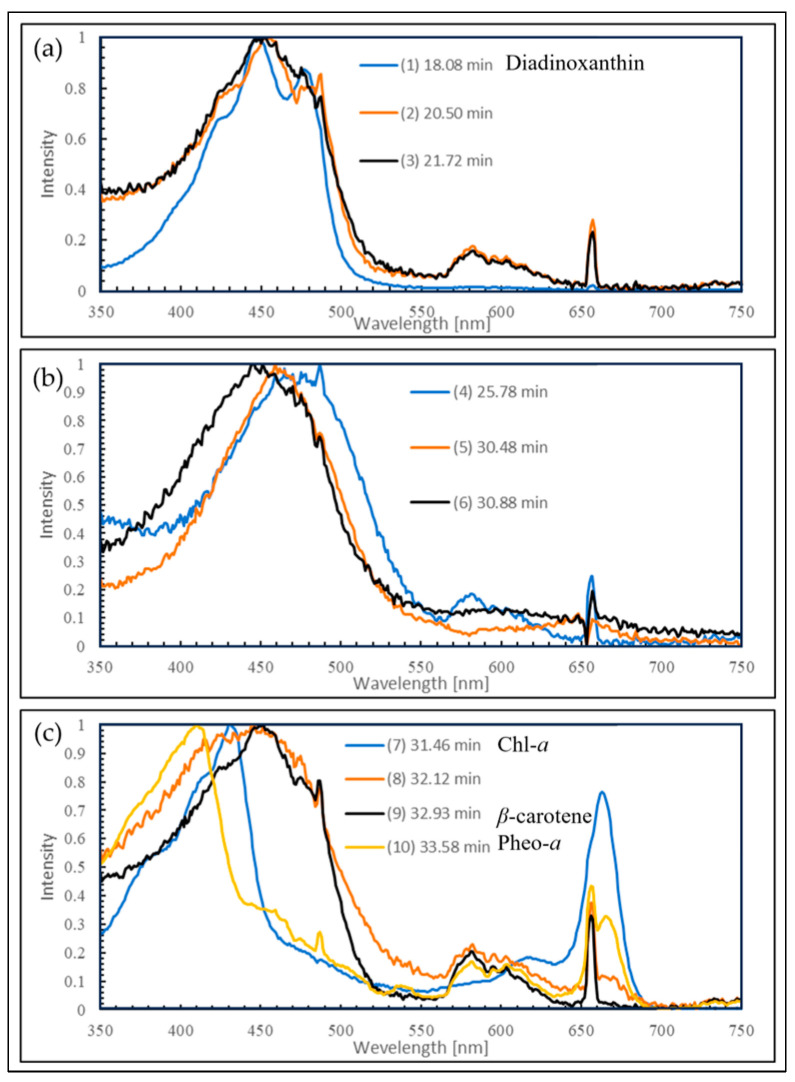
Absorption spectra and assignments for peaks corresponding to the elution times in Figure 11. (**a**–**c**) Each number corresponds to the number in Figure 11a.

**Figure 13:**
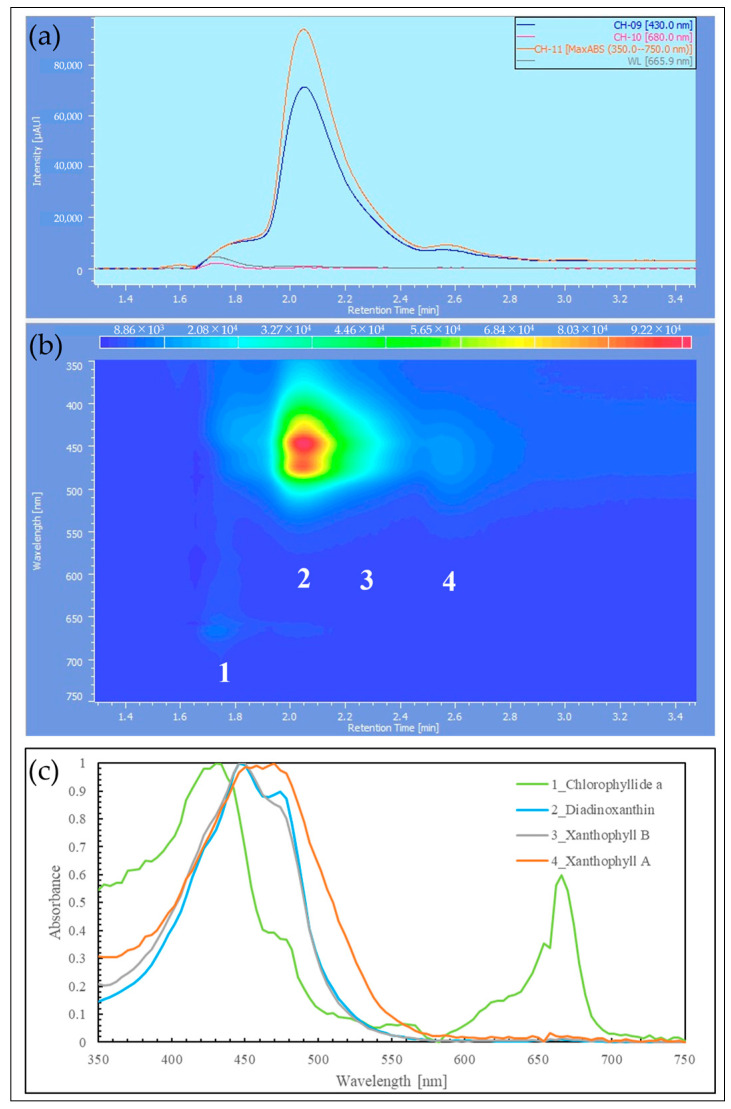
HPLC (Condition1) of reddened cells, low-polarity solvent extraction: (**a**) 2D chromatogram of cells reddened by strong red light irradiation in BS; (**b**) 3D chromatogram; (**c**) absorption spectra and assignments for peaks corresponding to elution times. Xanthophyll A, Xanthophyll B: unidentified xanthophylls; 2D chromatogram: the vertical axis in the upper diagram corresponds to absorbance, and the observation wavelength for the upper diagram is shown in the inset at the top right; 3D chromatogram: the vertical axis represents wavelength, and the Z-axis direction indicates absorbance changes through color variation.

**Figure 14:**
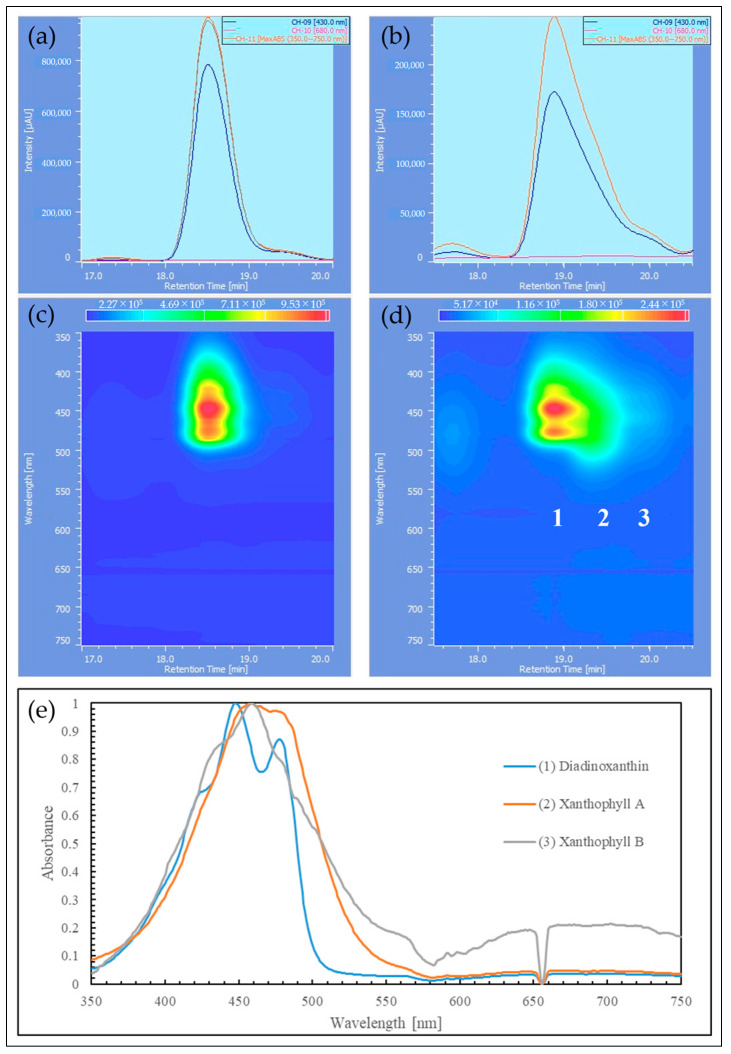
HPLC (Condition2), low-polarity solvent extraction: (**a**) 2D chromatogram of cells irradiated with normal fluorescent light in BS; (**b**) 2D chromatogram of cells reddened by strong red light irradiation in BS; (**c**) 3D chromatogram of (**a**); (**d**) 3D chromatogram of (**b**); (**e**) absorption spectra and assignments for peaks corresponding to elution times in (**b**). Xanthophyll A, Xanthophyll B: unidentified Xanthophylls; 2D chromatogram: the vertical axis in the upper diagram corresponds to absorbance, and the observation wavelength for the upper diagram is shown in the inset at the top right; 3D chromatogram: the vertical axis represents wavelength, and the Z-axis direction indicates absorbance changes through color variation.

The samples extracted with MeOH from the reddened cells and cells grown in CM medium were analyzed by HPLC, and their elution profiles were compared (Figure 8, Figure 9 and Figure 10). The HPLC method (Condition2), which allows for more detailed analysis of chlorophyll and carotenoids, was used to analyze the reddened cells (Figure 11 and Figure 12). Furthermore, when the extraction solvent was changed to decrease the polarity for the extraction, a similar elution pattern was obtained. The details around diadinoxanthin are presented in Figure 13 and Figure 14.

From Figure 8, it was observed that in the reddened cells, both Chl-*a* and Chl-*b* were significantly reduced. Also, in the reddened cells, peaks at 2.6 min and 6.0 min, which were not observed in cells grown in CM medium, were seen (Figure 8b). The peak that appeared at 2.6 min in the control was not present. The carotenoid peak at 6.0 min and the pheophytin *a* peak at 7.7 min increased.

Considering that diadinoxanthin is the most abundant xanthophyll in *E. gracilis* [51], the largest peak appearing just after 2 min is thought to be diadinoxanthin. The peak at 2.6 min had a broad absorption spectrum of xanthophylls extending to around 550 nm, similar to the red carotenoid pigment lycopene in tomatoes. The peak at 2.6 min is presumed to be a xanthophyll with a C=O bond, as it lacked the characteristic absorption maxima (0-0, 1-0, 2-0) of carotenes. The peak at 6.0 min, eluting after chlorophyll a, is likely to be a carotene similar to *β*-carotene.

From Figure 9b and Figure 10b, it is evident that the reddened cells contain a greater variety of substances (2.5~6.6 min, 350~570 nm).

From Figure 11a, it was observed that about ten peaks were detected using Zapata’s HPLC method. Compared to HPLC (Condition1), the detection intensity was reduced to about 1/5th, and the baseline rose with the increasing concentration of acetonitrile in the eluent B (Figure 11b). From Figure 12b, it is considered that Peak 4 corresponds to the 2.6 min mark in HPLC (Condition1) due to the shape of its spectrum (Figure 10c).

## 3. Discussion

The cell density of *E. gracilis* cultured in BS medium under white fluorescent light (90 µmol photons/m²/s, 30 °C) conditions reached more than four times that of cells cultured in CM medium on the seventh day (Figure 1). Moreover, in an experiment where growth was allowed until the stationary phase (Figure S1), the cell density in BS was more than five times that in CM on the eighth day, and the cell densities were comparable at the stationary phase, indicating that BS has a higher initial growth rate compared to CM. This indicates that the composition of the BS medium is conducive to the growth of *E. gracilis*. As the cell suspension is composed of the functional food BS and *E. gracilis*, it is considered to have a high nutritional value.

Histidine, a prominent component found in abundance in BS, is also an amino acid present in KH medium [42]. An investigation into whether reddening occurs in CM medium with the addition of an amount of histidine equivalent to that in KH medium, as well as with ten times that amount, showed no reddening. Additionally, culture mediums enriched with 5′-ribonucleotides (a mixture of inosinic and guanylic acids), which are umami components in seafood, and broths of dried sardine, cuttlefish, sakura shrimp, and shiitake mushrooms did not exhibit the vivid reddening of cell suspensions like BS. Hence, it is considered that the composition of various nutrients in BS influences this effect.

**Figure 15:**
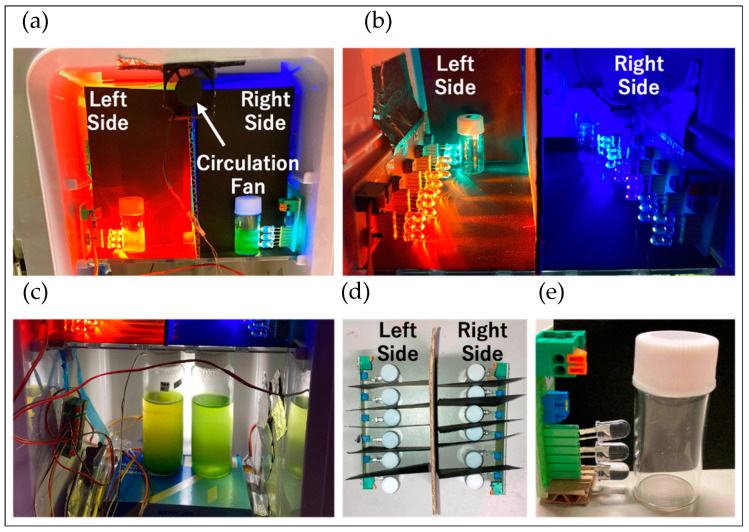
Incubator setup for LED cultivation: (**a**) Top section of the incubator. (**b**) Inside view of the top section of the incubator. (**c**) Bottom section of the incubator (left: BS medium, right: CM medium). (**d**) Top view of the inside of the top section of the incubator. (**e**) Arrangement of samples and LEDs (φ 5 mm round LEDs). The intensity of each of the three LEDs is listed in Table 2 under “each LED”.

**Figure 16:**
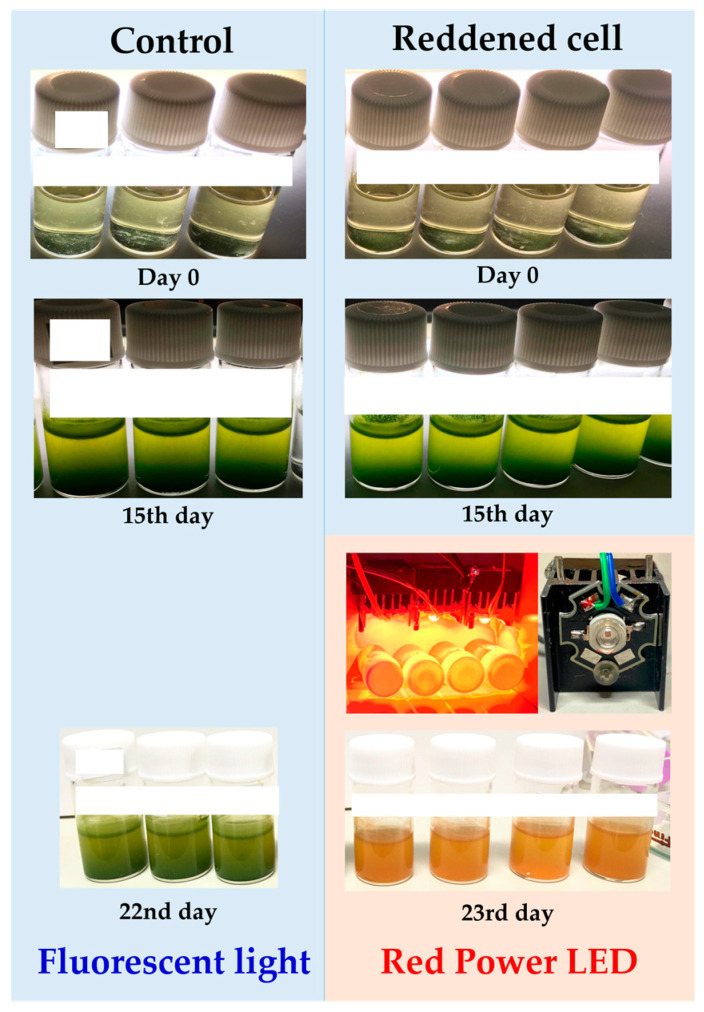
Temporal changes in culture solutions for HPLC measurement. Blue background: irradiated with fluorescent light. Red background: irradiated with red power LED. The samples in the bottom row were used for HPLC measurement.

Three experiments (June 2021, July 2021, November 2022) were conducted in this study (Figure 15), using the same brand of bonito flakes, though the batch for “November 2022” differed from the other experiments. In all cases, vivid reddening of the culture medium was confirmed (in chronological order of the experiments: Figure 7, Figure 16, and Figure 2). Particularly, as shown in Figure 16, the cell suspensions of four reddened samples under reddening conditions and three non-reddened samples under non-reddening conditions all showed similar color and intensity, indicating high reproducibility (for the reproducibility of single-cell absorption spectroscopy, please refer to Figures S2 and S3).

Regarding the absorption spectrum, both the cell suspension and single cells of *E. gracilis* cultured under white fluorescent light conditions (90 µmol photons/m^2^/s, 23 °C) in BS medium exhibited a slightly higher absorbance of carotenoids compared to the absorbance peak of Chl-*a* (675 nm) at 490 nm, relative to samples in CM and KH mediums (Figure 3 and Figure 5). Additionally, the shape of the Chl-*b* (620 nm) absorption peak in BS, for both cell suspension and single cells, was more indistinct compared to CM and KH. It is evident that the carotenoid content is higher relative to chlorophyll in *E. gracilis* grown in synthetic medium (Figure 4, “Control”).

In the exploration of conditions for reddening (medium, light wavelength, light amount, Table 2, Figure 2), significant visual reddening was confirmed in samples subjected to intense irradiation (1000~1300 µmol photons/m^2^/s, “M”, “H”) with warm wavelength LEDs (605~660 nm, Orange1~Red2) in BS medium. Conversely, under the same conditions, samples in CM medium exhibited a greenish-yellow color (yellowish green), with Orange1, 2 tending to be more yellow than Red1, 2. It is known that red light excites Chl-*a* in the photosynthetic center, activating the photosynthetic reaction, while blue light increases Chl-*b* (in *E. gracilis*, Chl-*b* is primarily found in proteins derived from PSII [52] and plays a role in preventing damage from excess light energy) [53].

In Figure 2, the closer the wavelength of the irradiated LED to the Q band absorption peak of Chl-*a*, the yellower the cell suspension in CM medium became, likely due to an increase in singlet oxygen produced by active photosynthesis, resulting in fewer maintainable chlorophylls.

Previous studies have reported that *E. gracilis* grown in CM medium mixed with 0.1% ethanol as a carbon source and exposed to continuous white light illumination at 920 µmol photons/m^2^/s results in a yellowish-green culture medium similar to that of CM medium [21]. In these studies, a decrease in chlorophyll content and the number of chloroplast thylakoid membranes was observed under strong light stress, indicating an increase in *β*-carotene, diadinoxanthin, diatoxanthin, and total carotenoids as a response to such light conditions [21]. Similar coloration of the culture medium was reported when grown in the same medium at low temperatures (20 °C) under continuous white light illumination at 55 or 240 µmol photons/m^2^/s [45]. In all these studies, an increase in the “diatoxanthin/diadinoxanthin” ratio was consistently observed, suggesting that the conversion from diadinoxanthin to diatoxanthin (i.e., the operation of the xanthophyll cycle) is a key factor in *Euglena*’s adaptation to light stress.

Considering that cell reddening in this study requires 1 to 2 weeks, it is believed to be due to carotenoid biosynthesis, differing from the mechanism seen in *E. sanguinea*, which reddens in less than 10 min not by new carotenoid generation but through rearrangement of cytoplasmic lipid droplets accumulated with astaxanthin [48]. Additionally, reddened cells revert to normal green cell suspensions within about a week when grown in CM medium under typical light conditions (white light, 90 µmol photons/m^2^/s). Therefore, cell reddening by this method is considered to be a non-genetically sustained defense mechanism against chronic light stress.

From Figure 3, samples in CM medium that exhibited a notable yellow color (Red1-M-CM, Red2-M-CM) were characterized by a decrease in the absorption peak of Chl-*a* (680 nm) and a flatter broad absorption peak of carotenoids (430~500 nm) compared to the control CM spectrum. In contrast, samples in BS medium showing significant reddening (samples with light amounts of M and H under various LEDs) had a significantly larger absorption peak of carotenoids (430~500 nm) compared to the absorption peak of Chl-*a* (680 nm). Furthermore, the absorption peak of carotenoids became larger towards shorter wavelengths, forming an ascending shoulder shape.

From Figure 4, it is observed that when the upward-sloping peak value of carotenoids (around 420 nm) becomes more than three times that of the absorption peak of Chl-*a* (680 nm), the culture medium visually appears red. On the other hand, samples in BS medium under the “L” light amount of various LEDs in Figure 3 showed a decrease in the “Carotenoid/Chl-*a*” absorption-peak intensity ratio and a dull green color mixed with red and green as the absorption peak value of Chl-*a* (680 nm) increased compared to samples under the “M, H” light amount. Additionally, from Figure 7, the results of measuring the absorbance in the short-wavelength region (250 nm ~) showed that samples grown in BS medium, regardless of the wavelength and intensity of irradiation light, had greater absorbance in the 260~370 nm range than samples in CM medium. Thus, the reddening of the culture medium is due to an increase in carotenoids relative to the content of Chl-*a*, suggesting that *E. gracilis* produces more types or amounts of carotenoids in BS medium than in CM medium.

From the Supplementary Information (Video S1), it was observed that in many cells reddened in CM medium, most of the Chl-*a* within the cells had faded. On the other hand, in cells that did not redden or in cells where chlorophylls and red pigments coexisted, the red regions were not as prominent. Also, the areas with chlorophylls were yellowish green, and cells that did not redden in BS (Red2-L-BS) appeared darker green, indicating the presence of more chloroplasts within these cells.

From the spectra of “Red2-M-CM1” and “Red2-L-BS” in Figure 5, it is clear that the latter has higher peak values of Chl-*a* (680 nm) and Chl-*b* (620 nm), indicating a higher content of these chlorophylls. In cells reddened in BS (Video S1, “Red2-M-BS”, and “Red2-H-BS”), many maintained a small amount of chlorophylls and showed recognizable red regions, unlike those in CM, which completely lost chlorophylls. Additionally, there were more motile, reddened cells in BS than in CM. This is likely because BS is a heterotrophic medium, allowing ATP synthesis without reliance on photosynthesis.

Reddened cells in *E. gracilis* have internal structures that exhibit red coloration, such as the eyespot and HC. The eyespot in *E. gracilis* is an organelle concentrated with carotenoids, including diadinoxanthin, diatoxanthin, and *β*-carotene [54,55]. HC, while resembling the eyespot in appearance, has a different absorption spectrum [30]. In Figure 6, data from our previous paper [30] have been recompiled to show these absorption spectra (“Eyespot(CM*)”, “HC(CM*)”). The absorption spectrum of HC is clearly different from that of the reddened cells. On the other hand, the spectrum of the eyespot shows a similar shape to that of the reddened cells, but with a gentler slope in the 520~580 nm range. Thus, the pigment composition of the reddened cells is suggested to be different from that of the eyespot.

Next, we consider the HPLC analysis results of the red substance. In *E. gracilis*, the most abundant carotenoid is diadinoxanthin, a type of xanthophyll, which constitutes 86% of the total carotenoids in the cell [21]. From this, the largest peak appearing just past 2 min in Figure 8, Figure 9 and Figure 10 is considered to be diadinoxanthin. Figure 8 reveals that in reddened cells, there was a significant decrease in Chl-*a* and Chl-*b* and an increase in pheophytin *a*. Additionally, new carotenoid peaks appeared at elution times of 2.58 and 5.99 min (Figure 10c). The peak at 2.58 min extended to around 550 nm on the long-wavelength side of the xanthophyll absorption spectrum, similar to the red pigment lycopene in tomatoes. The absence of the characteristic carotene absorption maxima (0-0, 1-0, 2-0) at this peak suggests it may be a xanthophyll with a C=O bond. The peak at 5.99 min, eluting after Chl-*a*, is presumed to be a carotene similar to *β*-carotene [51].

From Figure 11a, approximately ten peaks were observed using Zapata’s HPLC method. The peaks at each elution time are shown in Figure 12, and Peak 4, based on the shape of its spectrum, is thought to correspond to the 2.58 min elution peak in HPLC (Condition1) (Figure 10c). In the results where pigment samples extracted with low-polarity solvents were subjected to HPLC (Condition1, 2), no difference was observed in the HPLC elution patterns compared to the samples extracted with MeOH. Therefore, we will discuss the results of the enlarged area around diadinoxanthin in the elution profiles of each HPLC (Condition) (Figure 13 and Figure 14).

Figure 13 shows the results of reddened cells in HPLC (Condition1), where the elution peak 1 just before diadinoxanthin (elution peak 2) is thought to be Chlorophyllide-*a* (a decomposition product of Chl-*a* without the phytol chain) based on the shape of its absorption spectrum. From Figure 13c, there was a xanthophyll-like spectrum with an absorption peak on the longer wavelength side of diadinoxanthin. These are tentatively labeled as Xanthophyll A (elution peak 4) and Xanthophyll B (elution peak 3). These may be known or unknown xanthophylls that have not been identified in this study or their degradation products. Based on the absorption spectrum, Xanthophyll A is the elution peak specific to reddened cells in the HPLC (Condition1) analysis of the MeOH-extracted sample (Figure 10c, elution time: 2.58 min). Given its relative detection intensity, Xanthophyll A, which is present at about 1/10th the amount of diadinoxanthin, is believed to contribute to the red hue in reddened cells.

Figure 14 presents the results of HPLC (Condition2) for the control (cultured in BS medium, under white fluorescent light, at an intensity of 85 µmol photons/m^2^/s) and reddened cells. In the reddened cells, the peaks of Xanthophyll A (elution peak 2) and Xanthophyll B observed in Figure 13 were more prominent than in the control. Furthermore, compared to the intensity of the Chlorophyllide-*a* peak in the control (elution time: around 17.4 min), the peak in the reddened cells (elution time: around 17.7 min) was larger, suggesting a higher decomposition of Chl-*a* in the reddened cells.

Hence, when exposed to intense red light irradiation in BS medium, *E. gracilis* experiences enhanced decomposition and fading of Chl-*a* due to photoinhibition and, as a defense, maintains higher levels of diadinoxanthin contributing to the xanthophyll cycle and produces new Xanthophyll A. It is unclear whether the total carotenoid amount in the reddened cells increased compared to the control, as the total carotenoid content was not measured in this study. However, it has been reported that the total carotenoid content in cells generally increases under strong light stress [7,21,28], and there are reports of increased carotenoid production under low-temperature (20 °C) stress, suggesting that there may also be a temperature dependency [45]. Further verification of these findings remains a subject for future research.

The reddening of *E. gracilis* through this method is primarily related to the stress response. The metabolites produced by this process may affect the nutritional value and safety of the organism for human consumption. Therefore, it is crucial to analyze the composition and assess the safety of these metabolites before using them as edible products.

A method for optimizing biomass production using LEDs exists [56]. To efficiently produce carotenoids in *E. gracilis*, it is necessary to control the wavelength and intensity of LEDs. Additionally, it is important to investigate the impact of light-dark cycles on the reddening of *E. gracilis* to reduce LED irradiation time. Moreover, a light intensity of 1000 µmol photons/m^2^/s is necessary for *E. gracilis* reddening. This can be achieved by using filters that transmit only the red light wavelength band of sunlight. This suggests the possibility of large-scale outdoor cultivation in combination with LED lighting.

## 4. Materials and Methods

### 4.1. E. gracilis

The wild-type *E. gracilis* (Z strain) was provided by Euglena Co., Ltd. (Yokohama, Japan).

### 4.2. Cell Counting

Cell counting was performed using plankton counter plates (MPC-200, Matsunami Glass Ind., Ltd., Osaka, Japan) and a digital biological microscope (GR-D8T2, Shodensha, Inc., Osaka, Japan) with a 10× objective lens (NA 0.25, Shodensha, Inc., Osaka, Japan). During microscopic observation, the cell suspension was diluted and fixed with a solution made by diluting 10% (*w*/*v*) benzalkonium chloride solution (NIHON PHARMACEUTICAL CO., LTD., Osaka, Japan) 50 times with non-autoclaved purified water, resulting in a 0.2% (*w*/*v*) benzalkonium chloride solution. After all the cells had settled to the bottom of the plankton counter plates, they were counted.

### 4.3. For Exploring Cell Reddening Conditions (Influence of Wavelength and Light Intensity of LEDs and Culture Medium)

#### 4.3.1. Preparation of Bonito Stock Culture Medium

Distilled water was mixed with bonito flakes (Premium dried bonito: Marutomo Co., LTD., Ehime, Japan) to create a 3% (*w*/*w*) bonito stock solution. This solution was heated to maintain a boiling state for 1 min. After stopping the heat and allowing it to stand for 10 min, it was reheated to maintain boiling for another minute. Once cooled to room temperature, distilled water was added to replace the evaporated portion, maintaining a 3% (*w*/*w*) bonito stock solution. This was placed in a sealed container and stored in a refrigerator (4 °C) for 12 h, then filtered using Filter Paper No. 2 (Toyo Roshi Kaisha, Ltd., Tokyo, Japan). This filtrate was supplemented with V.B_12_ equivalent to KH medium and autoclaved.

The method for preparing samples for component analysis of “bonito dashi: bonito stock” in the “Standard Tables of Food Composition in Japan, 2015 Edition (Seventh Revision)” (17 Condiments and Spices) was referenced [57].

#### 4.3.2. Growth Curve in Bonito Stock Culture Medium

*E. gracilis* was aerobically cultured in a mixture of CM and KH media (CM:KH = 5:1; the mixing ratio was by eye measurement) for 19 days in an incubator at 30 °C. The lighting condition during this period was continuous irradiation at 30 µmol photons/m²/s using cold cathode tubes. 0.8 mL of this *E. gracilis* cell suspension was added to 80 mL of bonito dashi culture medium. As a control, the same volume of cell suspension, 0.8 mL, was added to 80 mL of CM medium. These samples were cultured for 7 days under the following conditions (Figure 15c):

Lighting condition: continuous irradiation with white fluorescent light;

Light intensity: 90 μmol photons/m^2^/s;

Temperature: 30 °C (incubator: Figure 15c);

Initial cell density: 2.1 × 10⁴ cells/mL.

Cell counting was performed daily during the 7-day culture period, and a growth curve was created (Figure 1).

#### 4.3.3. Confirmation of the Effect of Light Source Wavelength, Light Intensity, and Culture Medium on Cell Reddening

The cell suspensions cultured for 6 days were dispensed into 3 mL sterilized Laboran screw tube bottles (No. 2 (φ 18 × 40 mm), 6 mL, AS ONE Corporation, Osaka, Japan). The lids of each sample were closed loosely to ensure aerobic conditions, and the samples were placed in an incubator. The temperature inside the incubator was maintained at 30 °C, and a circulation fan was used to ensure uniform temperature distribution (Figure 15a). The door of the incubator was left slightly ajar (about 2~3 mm) to prevent biased gas composition inside. In the incubator, LEDs were aligned to irradiate each sample (Figure 15b). Adjacent samples were separated by black paper, and opposing samples were separated by cardboard to ensure each sample received the desired irradiation conditions (wavelength and light intensity) (Figure 15d).

Each sample was aligned with three LEDs of the same type, adjusted to nearly the same light intensity (Figure 15e). The detailed growth conditions for each sample are listed in Table 2. The LED light was continuously irradiated on the samples, and the appearances of each sample at the start of culture, and on the 5th and 15th days, are shown in Figure 2.

#### 4.3.4. Confirmation of Reddening through Absorbance Measurement

For the samples “Orange 1, 2” and “Red 1, 2”, which showed significant reddening as seen in Figure 2c, the absorbance of the cell suspension and individual cells was measured using the absorbance spectral imaging method. The measurement of the control sample was conducted separately from the series of experiments (4.3). The cultivation of the control sample is described below.

##### Cultivation of Control Sample

*E. gracilis* was aerobically cultured in a mixture of CM and KH media (CM:KH = 5:1; mixing ratio was by eye measurement) for 2 days in an incubator at 30 °C. The lighting condition during this period was continuous irradiation at 30 µmol photons/m²/s using cold cathode tubes. 20 µL of this *E. gracilis* cell suspension was dispensed into 3 mL of each of CM, KH, and BS media. These samples were cultured for 15 days under the following conditions (Figure 3, Figure 4, Figure 5 and Figure 6):

Lighting condition: continuous irradiation with white fluorescent light;

Light intensity: 90 μmol photons/m^2^/s;

Temperature: 23~25 °C;

Initial cell density: 4.2 × 10³ cells/mL.

For the measurement of cell suspensions, a glass cell with a path length of 5 mm was used, and for the measurement of individual cells, a glass-bottom dish and cover glass were used. Each sample was diluted to an appropriate concentration with fresh culture medium. The baseline for the measurement of cell suspensions was the absorbance of the fresh culture medium, and for the measurement of individual cells, the absorbance of the area with only the surrounding culture medium of the target cell was used.

##### Measurement of Absorption Spectra of Cell Suspensions and Individual Cells by Scan-Free Absorbance Spectral Imaging

Detailed methods of scan-free absorbance spectral imaging were previously described [46], and the subsequent improvement was reported [30,58,59]. Here, we give only brief specifications.

The system for scan-free (snapshot) absorbance spectral imaging is composed of an inverted microscope (IX71, OLYMPUS, Tokyo, Japan) and a custom-made fiber-bundled array to convert a 2-dimensional image to a 1-dimensional slit image. On the side port of the inverted microscope, the ×100 magnified image of the sample was focused on the fiber. The whole 3D image *A*(*x*, *y*, *λ*) is obtained (spatial resolution: about 1 × 1 μm^2^, wavelength resolution: 0.26 nm). The shortest acquisition time is 0.05 s.

For the absorbance measurement of cell suspensions, a glass cell with a path length of 5 mm was used, and for the absorbance measurement of individual cells, the glass bottom dish (Matsunami glass D11130H, Matsunami Glass Ind., Ltd., Osaka, Japan) was used, adequately diluted with fresh culture medium. Samples were set on the inverted microscope (IX71, OLYMPUS, Tokyo, Japan) and observed with the 100× objective lens of NA 0.85 (OLYMPUS, LCPlanFL N, Tokyo, Japan) from below. The light source was a 150 W Xenon lamp (Hamamatsu, Hamamatsu Photonics K.K., Shizuoka, Japan). The intensity (photon flux density), diameter of the sample, and exposure time are shown in Table 1.

For the measurement of cell suspensions, fresh culture medium was used as the baseline sample, and the microscope focus was adjusted near the surface of the glass cell (position where the focus is off the cells and only the uniform color of the culture medium is visible). The average absorbance of all areas in the obtained absorption image was calculated (Figure 3). Also, the ratio of the maximum absorption wavelength range for carotenoids (400~450 nm) to the absorption maximum of Chl-*a* (680 nm) was calculated and correlated with the appearances of each sample in Figure 2c and Figure 4.

In the measurement of individual cells, the baseline was set as the region where only the culture solution exists, and the microscope focus was adjusted to the target cell. The average absorbance of the red areas within reddened cells (excluding the eyespot) and the areas of high pigment concentration in green cells was calculated (Figure 5 and Figure 6). In both cases, the wavelength range was set from 395~750 nm, and the entire spectrum was adjusted by a constant value so that the absorbance at 750 nm became zero (Figure 3, Figure 5 and Figure 6).

### 4.4. Measurement of Absorption Spectrum of Cell Suspensions Using an Integrating Sphere

To clarify the absorption spectrum of cell suspensions in the shorter wavelength region without the influence of scattering by cells, absorbance measurement using the integrating sphere (IS) (inner diameter of 60 mm) mounted on a spectrophotometer (SolidSpec-3700DUV, Shimadzu Corporation, Tokyo, Japan) was performed. 600μL of cell suspension was placed in a custom-made cylindrical cell made of quartz glass (optical path length: 10 mm), and the optical cell was placed into the IS. For further details of the measurement method, please refer to our previous work [47]. The sample for baseline measurement was purified water, and absorbance measurement was carried out in the wavelength range of 250~750 nm (wavelength resolution 0.5 nm). The obtained spectra were adjusted by a constant value so that the absorbance at 750 nm was zero, and then normalized at the absorbance at 260 nm (Figure 7). Each sample was cultured under the following conditions (Table 3).

### 4.5. HPLC Analysis of Red Substance

#### 4.5.1. HPLC (Condition1) Analysis of Normal *E. gracilis* with MeOH Extraction

*E. gracilis* was aerobically cultured in CM medium under white fluorescent light (85 µmol photons/m²/s, 25 °C) until the stationary phase. 5 mL of this cell suspension was centrifuged (8000 rpm, 5 min, 2 °C), and the supernatant was removed. 500 µL of MeOH was added to the precipitate, vortexed, and then sonicated twice for 10 s each. This solution was centrifuged again (15,000 rpm, 5 min, 2 °C), and the supernatant passed through a 0.22 µm filter was used as the HPLC sample. HPLC was conducted under the following conditions:

HPLC (Condition 1)

Column: C8 (Senshu Scientific co., ltd., Tokyo, Japan)

Solvent: 98% MeOH, flow rate: 0.9 mL/min

Injection volume: 20 µL

The results are shown in Figure 8a and Figure 9.

#### 4.5.2. HPLC (Condition1) Analysis of Reddened E. gracilis with MeOH Extraction

*E. gracilis* was cultured for 15 days in BS medium (prepared as in Section 4.3.1) under white fluorescent light (85 µmol photons/m²/s, 25 °C), followed by 8 days of cultivation under Red PLED (1300 µmol photons/m²/s, 30 °C). Both light sources were continuously irradiated, and the culture was aerobically static for the entire period. Cultivation was carried out in four laboran screw tube bottles (No. 2) with 3 mL each, and all cell suspensions showed a similar degree of reddening (Figure 16, “Reddened cell”). On the 23rd day, all the culture media were mixed together as a sample for HPLC.

**Figure 17:**
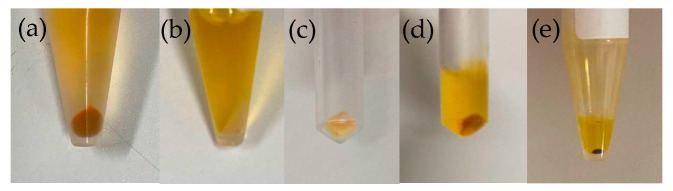
Preparation of samples for HPLC measurement (reddened cells). (**a**) Cell suspension after centrifugation. (**b**) Extract with 100% MeOH. (**c**) Precipitate after extraction. (**d**) Supernatant after cell disruption with glass beads. (**e**) Precipitate after concentration.

One milliliter of this reddened cell liquid was centrifuged (8000 rpm, 5 min, 2 °C) to precipitate the cells. The precipitate was reddish, and the supernatant was yellow (Figure 17a). After adding and mixing 1 mL of distilled water to this precipitate, it was centrifuged again (8000 rpm, 5 min, 2 °C), and the supernatant was removed. Pure MeOH 500 μL was added to the precipitate and vortexed, followed by sonication (10 s, 2 times) (Figure 17b). The precipitate post-color extraction was nearly white, but still had a slight reddish tint (Figure 17c). After the MeOH treatment, the cells were centrifuged (15,000 rpm, 5 min, 2 °C), and the methanol pigment fraction in the supernatant was passed through a 0.2 μm filter for HPLC (Supernatant of Figure 17b). HPLC (Condition1) was performed.

The results are shown in Figure 8b and Figure 10. Moreover, after the pigment extraction with MeOH, the yellow extract (supernatant of Figure 17b) was passed through a 0.22 µm filter, and after evaporating the solvent using an evaporator, 60 μL of 100% MeOH was added and concentrated, revealing a red precipitate (Figure 17e). This suggests that the red pigment was extracted in the yellow extract.

#### 4.5.3. HPLC (Condition2) Analysis of Reddened *E. gracilis* by Zapata’s Method

A method using a reverse-phase C8 column and a gradient of two different solvents (A, B solvent) to simultaneously separate chlorophylls and carotenoids was developed by Zapata. This method is known to exhibit good resolution for carotenoids, in addition to separating polar and non-polar chlorophylls [60]. The HPLC sample prepared in Section 4.5.2 was analyzed using HPLC under the following conditions.

HPLC (Condition 2)

A solvent: methanol/acetonitrile:0.25M aqueous pyridine (pH 5.0) = 50:25:25 (*v*:*v*:*v*);

B solvent: methanol/acetonitrile/acetone = 20:60:20 (*v*:*v*:*v*);

HPLC program (Table 4).

#### 4.5.4. Comparison of HPLC Elution Profiles of Samples Extracted with Low Polarity Solvent

Although 100% MeOH was used for the pigment extractions in Section 4.5.2 and Section 4.5.3, considering the possibility that the red pigment might be hydrophobic, the polarity of the extraction solvent was reduced by changing from 100% MeOH to a mixture of MeOH:Acetone:n-Hexane = 3:1:1 (*v*:*v*:*v*). After pigment extraction, concentration was carried out using an evaporator (30 °C, 30 min), followed by analysis using HPLC (Condition1, 2). However, the elution profiles for each HPLC (Condition1, 2) showed no differences compared to the results of tests with pigment extraction in 100% MeOH (Section 4.5.2 and Section 4.5.3).

Therefore, the elution profiles around diadinoxanthin in each HPLC condition were expanded and scrutinized. The results of HPLC (Condition1) for reddened cells are shown in Figure 13. The results of HPLC (Condition2) for the control sample and reddened cells are shown in Figure 14. The samples used here were reddened cells (prepared as in Section 4.5.2) and cells cultured in BS medium under white fluorescent light (control). The control sample was aerobically cultured under white fluorescent light (85 µmol photons/m²/s, 25 °C) for 22 days. They were cultured in three Laboran screw tube bottles (No. 2) with 3 mL each, and all cell suspensions showed a similar degree of green color. On the 22nd day, all culture media were mixed together as a sample for HPLC (Figure 16, “control”).

## 5. Conclusions

This study has for the first time identified the significant reddening conditions of *E. gracilis*, a *Euglena* species not previously thought to undergo reddening. The conditions involved using Bonito stock as the culture medium and irradiating with warm-wavelength LEDs (605~660 nm) at high light intensities (1000~1300 µmol photons/m²/s). This led to the decomposition of most chlorophylls in the cells due to excessive photosynthetic activity, while carotenoids, maintained to alleviate strong light stress, contributed to the formation of red-colored cells (Figure 4, Figure 6, and Figure 16). HPLC results revealed that the main component of these carotenoids was diadinoxanthin, and a previously unidentified xanthophyll A, not typically synthesized under regular growth conditions, was newly formed. Xanthophyll A is suggested to have C=O bonds from its absorption spectrum, and further detailed testing is necessary for its identification.

Regarding cultivation in BS medium, no reddening of cells was observed under general culture light conditions (white light, 90 µmol photons/m²/s), and the amount of Chl-*a* was less than in cells grown in standard synthetic medium. The cell density at the stationary phase was comparable to that in CM medium, but reached more than four times higher than CM medium on the 7th day of culture. This study is the first to report the use of animal-derived food products in the upcycling of *E. gracilis* from food and its by-products.

The cell suspension, consisting of the functional food BS and *E. gracilis*, is considered to have high nutritional value. Almost all cells underwent reddening when irradiated under conditions causing color change in BS medium. While reddening cells were also observed in CM medium, they accounted for only about 30% of the total, with the remaining cells showing a yellow-green color. Additionally, almost no motile cells were observed among those reddened in CM medium, whereas many of the reddened cells in BS medium were motile.

Adding histidine, a component unique to BS, to CM medium did not result in cell reddening, nor did the use of media made from four types of dried stock. This indicates that the nutrient composition in BS is crucial, and further testing is needed to identify several causative substances related to the color change. Generally, the total carotenoid content in cells is reported to increase under strong light stress. Since *E. gracilis* is known to increase carotenoid production under low-temperature (20 °C) stress, these aspects also remain subjects for future research. The insights obtained from this study and the clarification of these issues suggest the possibility of elucidating carotenoid production in *E. gracilis* and its potential for industrial use.

## Figures and Tables

**Table 1:** Conditions for absorbance measurement.

Sample	Incubation Period [Day]	Intensity [µmol Photons/m^2^/s]	Light Diameter [mm]	Exposure Time [s]
CM, KH, BS	14	7400	3.0	0.3
Orange 1, 2	16	7100	3.5	0.4
Red 1, 2	15	7700	3.5	0.4

**Table 2:** Relationship between light source wavelength and intensity (Figure 2).

LED Color	Wavelength [nm]	Intensity (Symbol)	Intensity [µmol photons/m^2^/s] Average (Each LED)	Culture Medium
Dark	-	-	0 (-)	CM
		-	0 (-)	BS
Ultraviolet	365	-	10 (9, 11, 10)	BS
	375	-	21 (19, 25, 20)	BS
	395	-	290 (270, 276, 325)	BS
	405	L	496 (470, 542, 476)	BS
Blue	475	M	1029 (978, 1140, 969)	CM
		M	1008 (912, 1074, 1037)	BS
Green	505	M	1029 (1040, 1044, 1002)	CM
		M	1033 (1004, 1062, 1033)	BS
Yellow	590	M	508 (482, 538, 503)	CM
		M	495 (477, 530, 478)	BS
Orange1	605	M	1027 (963, 1108, 1010)	CM
		L	495 (486, 510, 490)	BS
		M	1031 (987, 1054, 1051)	BS
		H	1282 (1256, 1315, 1275)	BS
Orange2	611	M	980 (964, 1036, 940)	CM
		L	496 (486, 511, 491)	BS
		M	1004 (980, 1043, 990)	BS
		H	1282 (1262, 1367, 1218)	BS
Red1	625	M	1012 (1036, 1035, 965)	CM
		L	494 (456, 510, 517)	BS
		M	1015 (999, 1020, 1025)	BS
		H	1337 (1297, 1336, 1378)	BS
Red2	660	M	1012 (1023, 1023, 990)	CM
		L	490 (504, 514, 451)	BS
		M	1002 (975, 1005, 1025)	BS
		H	1299 (1214, 1368, 1314)	BS
White	-	L	514 (494, 536, 513)	CM
		L	502 (485, 536, 484)	BS

L: low intensity; M: middle intensity; H: high intensity. (Each LED): The intensity of each of the three LEDs in Figure 15e. CM: CM medium; BS: Bonito stock medium.

**Table 3:** Culture conditions.

Sample	Culture Medium	Incubation Period [Day]	Light Source	Intensity [µmol Photons/m^2^/s]	Temperature [℃]
CM	CM	18	White fluorescent light	90	25
BS (FL)	BS*	33	White fluorescent light	90	25
BS (Red-PLED)	BS*	33	Red Power LED (625 nm)	1300	25

BS*: Prepared according to the method in Section 4.3.1, but with a 0.9% (*w*/*w*) bonito stock solution instead of the 3% (*w*/*w*) bonito stock solution. All samples were continuously illuminated with culture light and aerobically incubated in a static state. The cell suspension and microscopic photographs on the 29th day of the BS (Red-PLED) are in Supplementary Materials Figure S1, and the results of the single-cell absorbance measurement are in Figure S2.

**Table 4:** HPLC program.

Min	B Solvent [%]
0	0
22	40
28	95
38	95
40	100

0.9 mL/min liner gradient.

## Data Availability

Data are contained within the article and its Supplementary Materials.

## Supplementary Materials

The following supporting information can be downloaded at: https://www.mdpi.com/2223-7747/13/4/510/s1, Figure S1: Growth curve of *E. gracilis* under typical cultivation conditions; Figure S2: Microscopic images of the reddened culture medium and cells; Figure S3: Single cell absorbance spectra of reddened cells; Table S1. Conditions for measurement of absorption spectral images (“Cell1~3” in Figure S3); Video S1: Microscopic movie of reddened cells.
