# Supplementary material for: Reddening of the Unicellular Green Alga Euglena gracilis by Dried Bonito Stock and Intense Red Light Irradiation
Source: Plants (Basel). 2024 Feb 12;13(4):510. doi: 10.3390/plants13040510 (PMC10892402; doi:10.3390/plants13040510)
Supplement: Supplementary file 1 [file plants-13-00510-s001.zip › plants-2820148-supplementary.pdf]

Supplementary information

1. Growth curve in Bonito stock (BS) medium until stationary phase

This data is a re-edited version of the growth curve presented in our paper [1], where the growth curve (originally showing only CM and KH) has been supplemented with BS data.

Although the experimental conditions slightly differ from those described in the main text (Figure 1), such as culture volume, temperature, and stirring during cell counting, the cell density in BS at day 8 in Figure S1 was more than five times higher than that in CM. This trend is similar to the one observed on day 7 in Figure 1 of the main text, where the cell density in BS was more than four times that of CM. On the same day, the cell density in BS was 0.7 times that in KH. After day 12, the cell densities in CM and BS became comparable, indicating that BS has a higher initial growth rate.

Cell counting was performed from day 8 to day 14 after the start of culture to generate a growth curve (Figure S1).

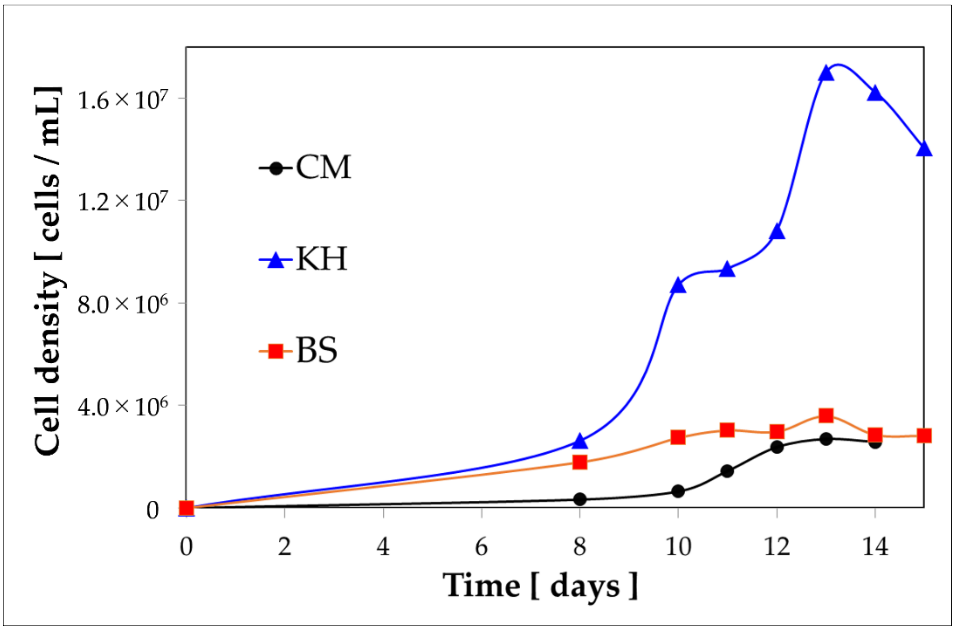

Figure S1. Growth curve of *E. gracilis* under typical cultivation conditions

2. Single-cell absorbance spectrum of *E. gracilis* cultured in BS medium under red light high-intensity irradiation conditions

E. gracilis was cultured for 29 days in BS medium under red light high-intensity irradiation, and the reddening of both the cell suspension and the cells was confirmed (Figure S2). The thinning of the green color of the cells due to the decolorization of chlorophylls made the red pigments, presumed to be carotenoids, distinctly observable. For detailed culture conditions, please refer to "4.4. Absorbance spectrum measurement of cell suspension using an integrating sphere" in the main text.

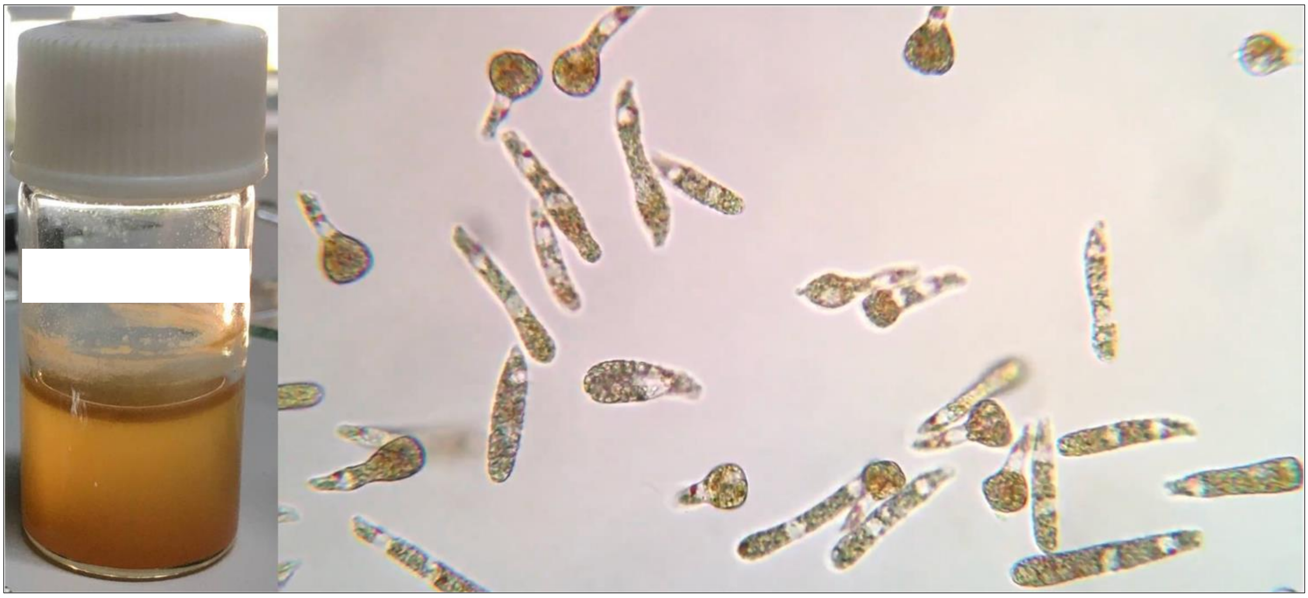

Figure S2. Microscopic image of the reddened culture medium and cells

The absorption spectra of the above single cells were measured and compared with the spectra of the reddened single cells in Figure 6 of the main text (Figure S3).

Please refer to section 4.3.4.2 in the main text for the method of absorption spectrophotometry and analysis. The conditions for absorption spectroscopic imaging measurements are also described in that section. The measurements of “Cell1~3” in Figure S3 were conducted under the conditions listed in Table S1.

Table S1. Conditions for measurement of absorption spectral images (“Cell1~3” in Figure S3)

**Incubation period [day]**	**Intensity [μmol photons/ m²/sec]**	**Light diameter [mm]**	**Exposure time [sec]**
29	1370	3.0	0.5

"Cell1" and "Cell3 (Near eyespot)" exhibited spectra similar to the "Red2-H-BS" cells shown in Figure 6. The dividing reddened cell, "Cell2", displayed a lower absorbance in the 570~659 nm range compared to the aforementioned cells. "Cell3 (Eyespot)" had a spectral shape similar to the "CM* (Eyespot)" in Figure 6. "Cell1~3" were cells in the culture medium that had been continuously exposed to intense red light irradiation right after subculturing, and they yielded absorbance spectra similar to those seen in Figure 6.

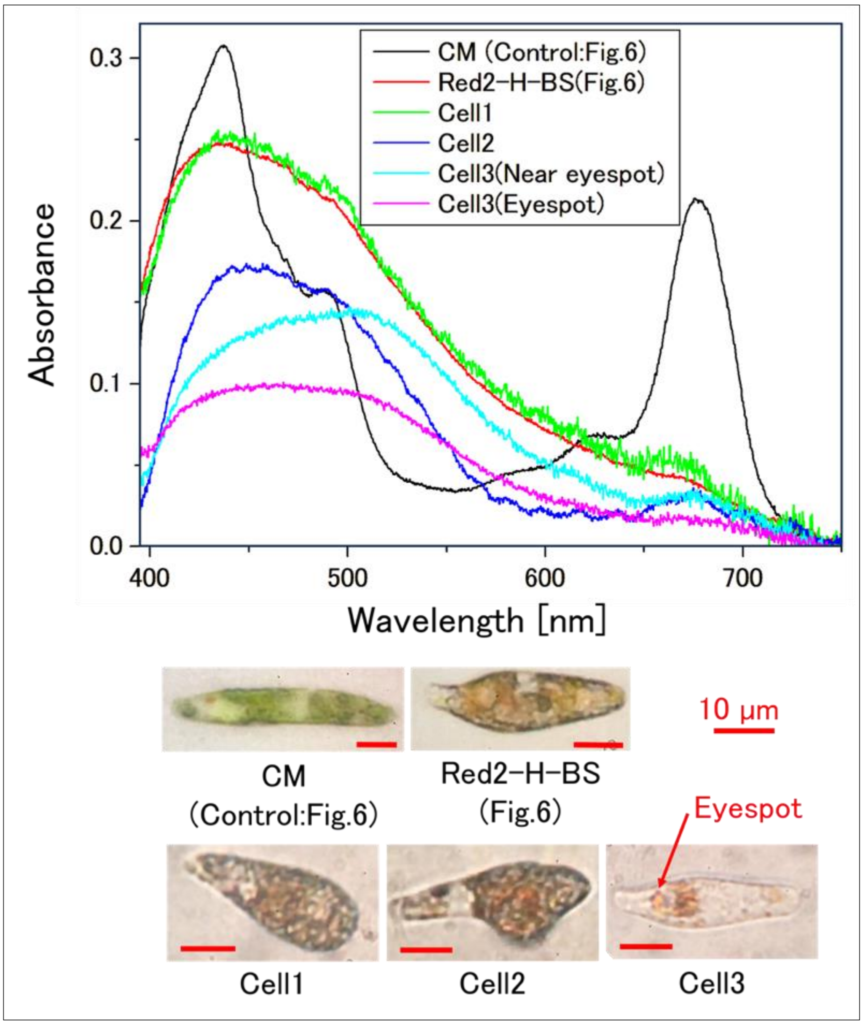

Figure S3. Single-cell absorbance spectra of reddened cells.

・Data and photographs for "CM" and "Red2-H-BS" are referenced from Figure 6 in the main text.

・"Cell1~3" are from the culture medium shown in Figure S1.

・Cell1: Reddened cell.

・Cell2: Cell undergoing division.

・Cell3: Cell with almost completely faded chlorophylls.

Reference

1. Yamashita, K.; Yamada, K.; Suzuki, K.; Tokunaga, E. Method for Growing Edible *Euglena gracilis* in an Inexpensive Medium with Tomato Juice to a High Cell Density Equivalent to the Density in KH Medium. *Sustain. Food Technol.* **2023**, *1*, 709–721, doi:10.1039/D3FB00086A.
